# Early Lyme disease – current clinical and epidemiological aspects in Braşov

**DOI:** 10.1186/1471-2334-13-S1-P63

**Published:** 2013-12-16

**Authors:** Maria Elena Cocuz, Ligia Rodina, Iuliu Gabriel Cocuz

**Affiliations:** 1 Faculty of Medicine, Transilvania University, Braşov, Romania; 2 Infectious Diseases Hospital, Braşov, Romania; 3 Faculty of Medicine, University of Medicine and Pharmacy Tîrgu Mureş, Romania

## Background

Lyme disease, endemic in Europe, North America and Asia, is caused by the *Borrelia burgdorferi* bacteria. In the absence of a correct diagnosis and an early and appropriate treatment, it evolves in 3 stages: early localized stage (erythema migrans – present in the majority but not in all the patients – fever, fatigue, myalgia, headache, neck stiffness); early disseminated stage (secondary skin lesions and extra cutaneous manifestations, like muscular/skeletal and hematologic symptoms, less common cardiac disturbance); chronic disseminated stage (rheumatological and neurological manifestations, acrodermatitis chronica atrophicans). The aim of this study was to analyze some epidemiological and clinical aspects in the early stage of Lyme disease in patients admitted into the Infectious Diseases Hospital of Braşov.

## Methods

The study was performed retrospectively on 56 patients admitted to the Infectious Diseases Hospital in Braşov during 2009-2012, in which Lyme disease was confirmed based on epidemiologic, clinic and serologic (the presence of anti-*Borrelia burgdorferi* IgM) findings. The epidemiological aspects analyzed were dynamic and seasonality of admissions, sex, age and area of origin of the patients. Clinically, we assessed the presence of erythema migrans and other clinical manifestations, and paraclinically, nonspecific laboratory data: ESR, serum fibrinogen and C-reactive protein changed data.

## Results

During the analyzed period of time, the number of admissions for Lyme disease has increased (from 11 cases in 2009 to 19 cases in 2012). The frequency of the disease was equal in both sexes, 48 (85.71%) patients were adults and 51 (91.07%) were from urban areas; most often, the hospital admission occurred in May, June and July (50%). Erythema migrans was diagnosed in 43 (77%) patients; other clinical manifestations were fever and chills (30.36%), headache (12.5%) and arthralgia (12.5%). Increases of ESR, fibrinogen and CRP serum values have been highlighted in 30.36%, 23.21% and 32.14% of cases.

## Conclusion

Lyme disease shows persistent and growing morbidity. Admissions for Lyme disease prevailed in the hot season in adult patients in urban areas. Erythema migrans was present only in 3/4 of the patients, while other symptoms (uncharacteristic) were rare. Biological inflammatory syndrome was seen only in up to one third of patients.

